# Molecular Orientation of Carboxylate Anions at the Water–Air Interface Studied with Heterodyne-Detected Vibrational Sum-Frequency Generation

**DOI:** 10.1021/acs.jpcb.2c08992

**Published:** 2023-03-14

**Authors:** Alexander A. Korotkevich, Carolyn J. Moll, Jan Versluis, Huib J. Bakker

**Affiliations:** Ultrafast Spectroscopy, AMOLF, Science Park 104, Amsterdam 1098XG, Netherlands

## Abstract

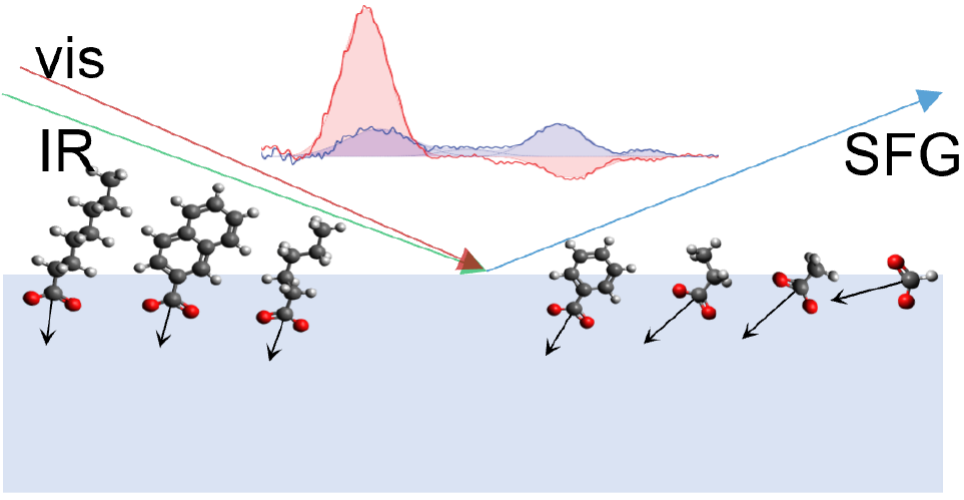

The carboxylate anion group plays an important role in many (bio)chemical systems and polymeric materials. In this work, we study the orientation of carboxylate anions with various aliphatic and aromatic substituents at the water–air interface by probing the carboxylate stretch vibrations with heterodyne-detected vibrational sum-frequency generation spectroscopy in different polarization configurations. We find that carboxylate groups with small aliphatic substituents show a large tilt angle with respect to the surface normal and that this angle decreases with increasing size of the substituent. We further use the information about the orientation of the carboxylate group to determine the hyperpolarizability components of this group.

## Introduction

**Figure 1:**
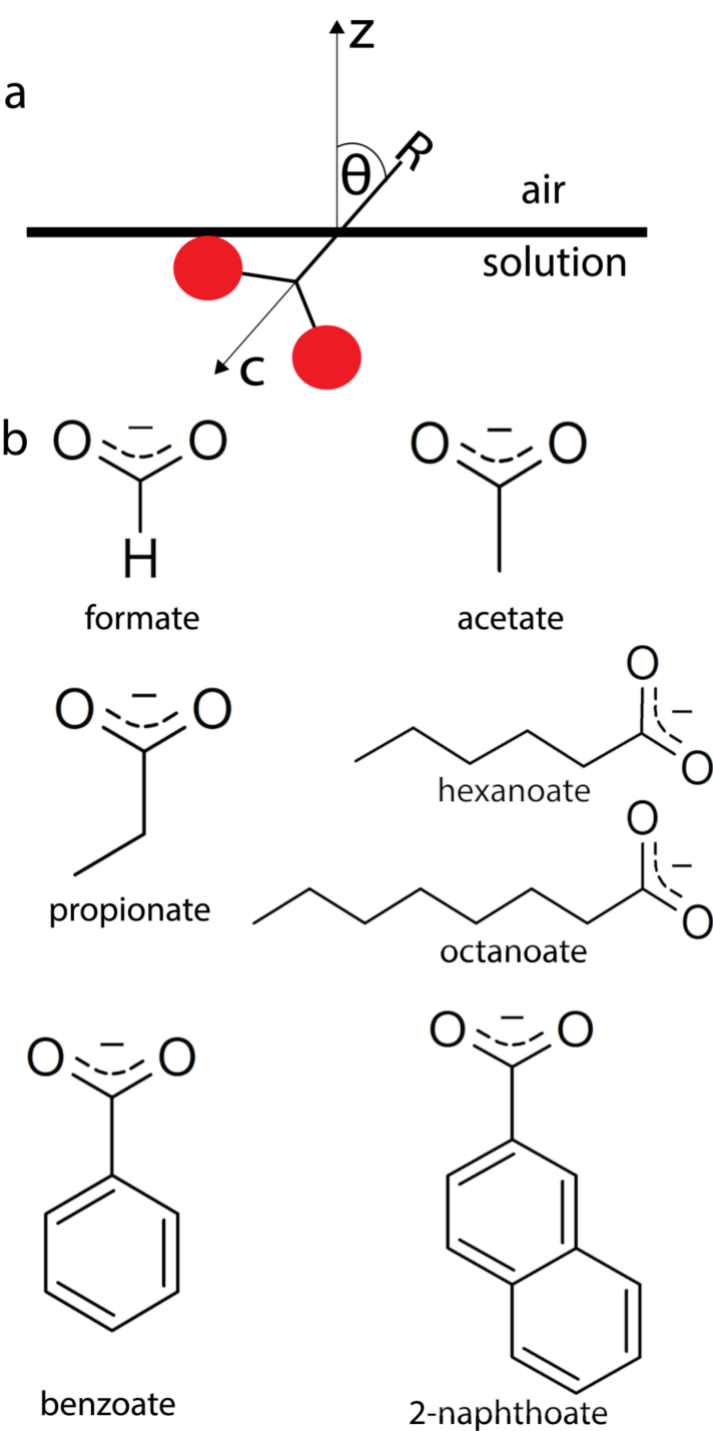
(a) Definition of the tilt angle θ; red spheres represent oxygen atoms of a carboxylate anion. (b) Chemical structures of the carboxylates under study.

Carboxylic acids and carboxylate anions are abundant in biological and abiotic systems and are ubiquitous in organic synthesis reactions and industrial applications.^1−10^ Both species can be located at aqueous interfaces, dependent on their overall hydrophobic/hydrophilic character, the subphase pH, and the ionic strength. Previous studies have focused on the degree of surface adsorption, the surface p*K*_a_ of carboxylic acids,^11−13^ the interaction of Langmuir–Blodgett monolayers of long-chain fatty acids with metal cations,^14−16^ and the mechanisms of emulsion stabilization by carboxylic acid/carboxylate surfactants.^17^ An important property of carboxylate ions and carboxylic acids at the water–air interface concerns their orientation, as this property impacts their spatial charge distribution and solvation structure, which play important roles in atmospheric processes, chemistry of aerosols, and soil chemistry. Vibrational sum frequency generation spectroscopy (VSFG) is an efficient tool for investigating interfacial molecules and ions. This technique is highly surface-specific and enables the characterization of the adsorption and orientational properties of species adsorbed at a phase boundary via their vibrational response. The growing interest in the molecular orientation and adsorption of molecules and ions at interfaces stimulates VSFG studies of various interfaces relevant for material design and biochemistry.^18−24^ Previous VSFG studies have focused on the orientation of interfacial water molecules at neutral and charged interfaces,^23,25−27^ the structure of biodegradable polymers^28^ and proteins,^29^ and the adsorption of small molecules^30−33^ and surfactants.^34,35^ VSFG spectroscopy has been used to study the orientation of formic, acetic, and hexanoic acids at the water–air interface.^36−38^ The technique has also been used to study the orientation of formate and acetate anions probing the symmetric stretch vibration (ν_s_) and the antisymmetric stretch vibration (ν_as_) of the carboxylate group.^39^ The measured signals allowed for an estimation of the tilt angle θ of these species at the water–air interface.^39^ The tilt angle θ is defined as the angle between the molecular *c*-axis and the laboratory *z*-axis, as shown in Figure 1a. The average tilt angle of the acetate ion was found to be ∼45°, while that of the formate ion was found to be close to 90°. These results appear to be in contrast with previous molecular dynamics (MD) simulations that indicated, for acetate and benzoate ions, the tilt angle would show a distribution with its maximum at ∼0° and a full width at half-maximum (FWHM) of ∼50–60°. These observations stimulate further exploration of surface adsorption and the dependence of the tilt angle of carboxylate anions on the size and structure of the substituent. In this work, we study the molecular orientation at the water–air interface of carboxylate anions with different structures of the substituents. In Figure 1 we show structural formulas of the carboxylate anions investigated in this work. The studied anions can be divided into two groups. The first group includes ions with aliphatic chains with a different number of carbon atoms: formate, acetate, propionate, hexanoate, and octanoate. The second group includes aromatic species with different structures: benzoate and 2-naphthoate. We probe the response of the ν_as_ carboxylate stretch vibration with heterodyne-detected vibrational sum frequency generation (HD-VSFG) spectroscopy in different polarization configurations. We combine this information with the measured responses of the ν_s_ carboxylate stretch vibration, which allows us to determine the relation among several of the hyperpolarizability components of the carboxylate anion group.

## Materials and Methods

### Sample Preparation

We used sodium carboxylates as received: formate (≥99.0%, Fluka Analytical), acetate (anhydrous, for molecular biology ≥99%, Sigma-Aldrich), propionate (minimum 99%, Sigma), hexanoate (99–100%, Sigma), octanoate (≥99%, Sigma), benzoate (>99%, Sigma-Aldrich), and 2-naphthoate (>98%, TCI). To prepare the samples, we dissolved appropriate amounts of the salts in D_2_O (99.9% D atom, Aldrich). For each carboxylate, we chose the concentration to obtain a signal-to-noise ratio that is more than sufficient to reliably determine the amplitudes of the VSFG responses of the ν_s_ and ν_as_ vibrations of the carboxylate groups in different VSFG polarization combinations. The concentrations of the solutes are reported in the units of molality (mol/kg solvent (m)).

### FTIR Measurements

We measured the infrared absorption spectra of all samples with a Bruker Vertex 80v Fourier-transform infrared spectrometer with a resolution of 2 cm^–1^. The samples were prepared by squeezing a droplet of solution between two circular 1 mm thick CaF_2_ windows (Crystran) without using spacers. An empty cell containing a 2 mm thick CaF_2_ window is used for background subtraction.

### Heterodyne-Detected Vibrational Sum-Frequency Generation Spectroscopy

The HD-VSFG measurements were performed with a home-built setup based on a commercial Ti-sapphire regenerative amplifier (Coherent Legend Duo) seeded by a commercial oscillator (Coherent Mantis). The amplifier delivers ∼35 fs long pulses with an energy of ∼6 mJ per pulse at a 1 kHz repetition rate. The output of the amplifier is split into two parts. The first part is sent to a pulse-shaper in order to generate a spectrally narrow (∼20 cm^–1^) 800 nm pulse (ω_vis_). The second part of the output is sent to a commercial optical parametric amplifier and difference-frequency mixing stage (Light Conversion HE-TOPAS) to generate ∼400 cm^–1^ infrared pulses centered at ∼1550 cm^–1^ (ω_IR_). The laser and parametric generation device deliver s-polarized ω_vis_ and p-polarized ω_IR_ light, respectively. To obtain p-polarized ω_vis_ and s-polarized ω_IR_ we use two half-wave plates (λ/2) to rotate the polarization direction and cleaning polarizers. The energies of the ω_vis_ and ω_IR_ pulses are ∼20 and ∼15 μJ, respectively. The ω_vis_ and ω_IR_ beams are focused and overlapped on the surface of a gold mirror to generate a local oscillator sum-frequency generation signal (LO-SFG, ω_SFG_ = ω_vis_ + ω_IR_). The LO-SFG light is sent through a 1 mm thick silica plate to delay it by ∼1.6 ps with respect to the ω_vis_ and ω_IR_ beams. All of the beams are refocused on the sample surface, where the ω_vis_ and ω_IR_ beams generate the sample sum-frequency generation signal. The sample SFG and the LO-SFG are then sent to a spectrometer, and their intensity is detected frequency-resolved by a thermoelectrically cooled charged-coupled device (CCD, Princeton Instruments). The sample SFG and the LO-SFG interfere, and from the interference pattern, the real and imaginary parts of the χ^(2)^ spectrum of the sample can be extracted, which contains information about the orientation of the molecular functional groups. The dependence of the signal on the intensity spectrum of the IR pulse is divided out by repeating the experiment with a reference z-cut α-quartz instead of the sample. The height of the α-quartz surface is carefully adjusted to that of the sample surface to minimize the error in determining the phase of the sample SFG signal. The phase uncertainty of the experiments is smaller than π/10 . The sample spectra are obtained by averaging five scans with an acquisition time of 120 s for each scan. In the 1300–1650 cm^–1^ frequency region a strong etalon effect occurs in the CCD camera, which can significantly distort the spectra. This effect is corrected by taking two reference spectra from the quartz crystal. The phases of the two spectra differ by 180°, which is achieved by rotating the quartz crystal by 180° around its *z*-axis. The details of this correction procedure have been reported before.^27,39^

## Theoretical Background

To extract information about the orientation of the carboxylate ions at the surface, we determine the peak amplitude of the ν_as_ vibration from the Im[χ^(2)^] spectra collected in SSP and SPS polarization combinations, where the notation SS(P)P(S) refers to S-polarized light at ω_SFG_, S(P)-polarized light at ω_vis_, and P(S)-polarized light at ω_IR_. The expressions connecting the experimentally determined χ^(2)^ components and the orientational properties for molecular groups belonging to the *C*_2*v*_ symmetry group have been derived before.^40^ In this derivation, free rotation of the carboxylate group around the neighboring C–C (C–H) bond is assumed. For ν_as_ and ν_s_ vibrations in SSP and SPS polarization combinations, the following expressions have been obtained.
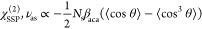
1
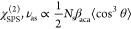
2
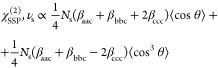
3
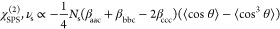
4

In these expressions, *N*_s_ is the surface density, β_*ijk*_ are the hyperpolarizability components with the indices *i*, *j*, and *k* referring to the molecular axes *a*, *b*, and *c*, and θ is the tilt angle between the molecular *c*-axis and the surface normal, as shown in Figure 1a.

Dividing eq 1 by eq 2 we obtain
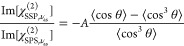
5

As follows from eq 5, the tilt angle θ can be determined directly from the ratio of the amplitudes of the responses of the ν_as_ vibration in the SSP and SPS Im[χ^(2)^] spectra. The coefficient *A* is introduced to account for the ratio of the Fresnel coefficients and the ratio of the sines of the incidence angles of ω_IR_ and ω_vis_ beams, as described in the Supporting Information. The calculation of the cosine terms on the right-hand side of eq 5 includes integration over the angular distribution of the carboxylate anion.

## Results and Analysis

**Figure 2:**
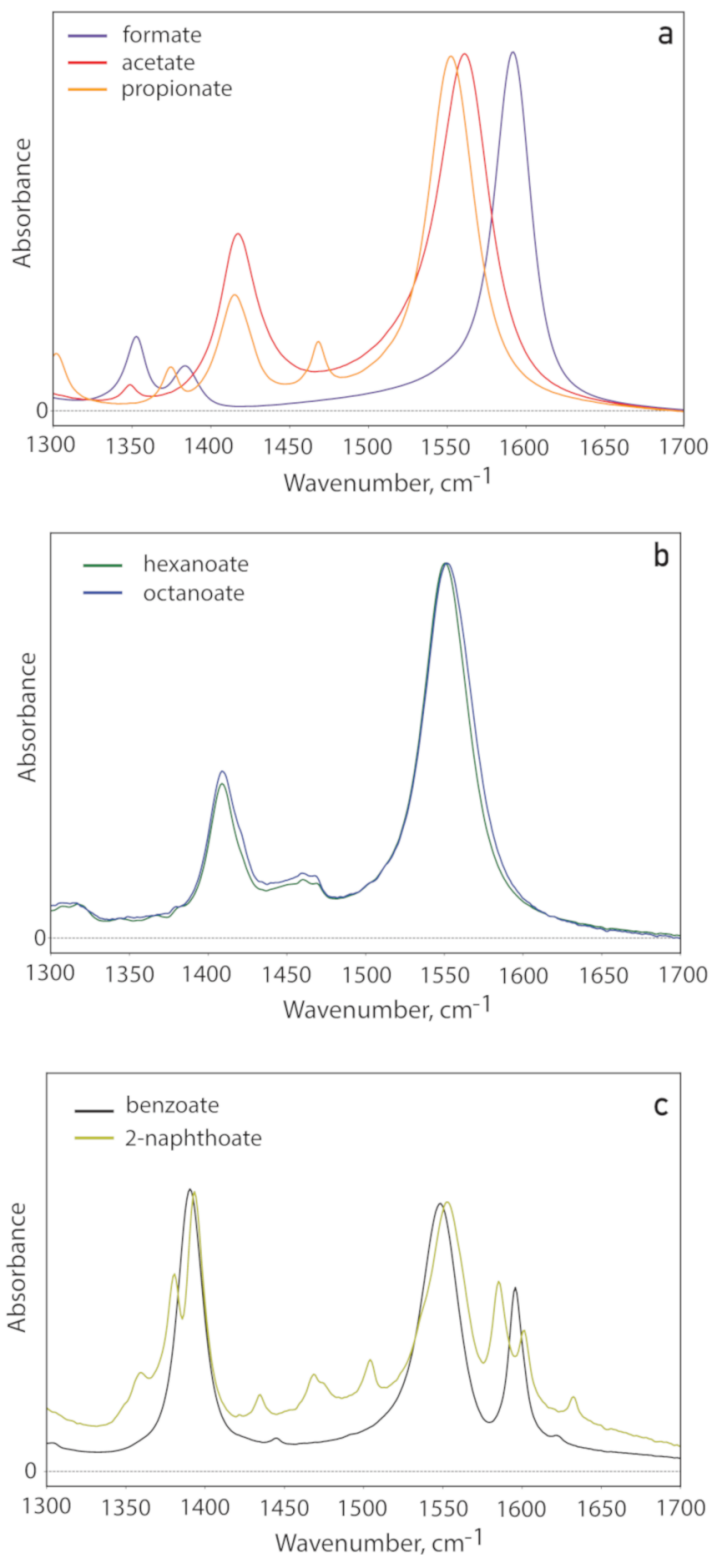
Infrared absorption spectra of 1 m sodium carboxylates (a) formate (purple), acetate (red), propionate (orange), (b) hexanoate (green), octanoate (blue), and (c) benzoate (black), 2-naphthoate (khaki). The spectra are normalized with respect to the amplitude of the band corresponding to the ν_as_ vibration.

In Figure 2 we show linear infrared absorption spectra of the studied carboxylates in the 6 μm region. The spectra of formate, acetate, and propionate are shown in Figure 2a. We assign the band centered at 1352 cm^–1^ for formate, 1417 cm^–1^ for acetate, and 1415 cm^–1^ for propionate to the ν_s_ vibration of the carboxylate group. The strong absorption band centered at 1593 cm^–1^ for formate, at 1561 cm^–1^ for acetate, and at 1553 cm^–1^ for propionate is assigned to the ν_as_ vibration of the carboxylate group. In addition, for formate, we assign the band centered at 1383 cm^–1^ to the rocking vibration of the methine CH group. For acetate and propionate, we assign the bands centered at 1348 and 1374 cm^–1^ to the symmetric bending vibrations of the CH_3_ group . Lastly, for propionate we assign the band centered at 1468 cm^–1^ to the antisymmetric bending mode of the CH_3_ group .^41−43^

As can be seen in Figure 2b, the spectra of hexanoate and octanoate are almost identical. Similarly to the propionate ion, we assign the bands centered at 1412 and 1550 cm^–1^ to the ν_s_ and ν_as_ vibrations, respectively, and the band centered at 1462 cm^–1^ to the  mode.

For both benzoate and 2-naphthoate the bands corresponding to ν_s_ and ν_as_ vibrations are centered at 1390 and 1550 cm^–1^, respectively, and have very similar resonance frequencies, as shown in Figure 2c. For the benzoate ion, the band centered at 1596 cm^–1^ corresponds to the ring mode of the ion. This band is relatively strong and is thus likely of mixed character, borrowing oscillator strength from the ν_as_ vibration.^44^ 2-Naphthoate shows a plethora of weaker absorption bands, resulting from the coupling of the vibrations of the two-ring aromatic system with the ν_s_ and ν_as_ modes of the COO^–^ group.

**Figure 3:**
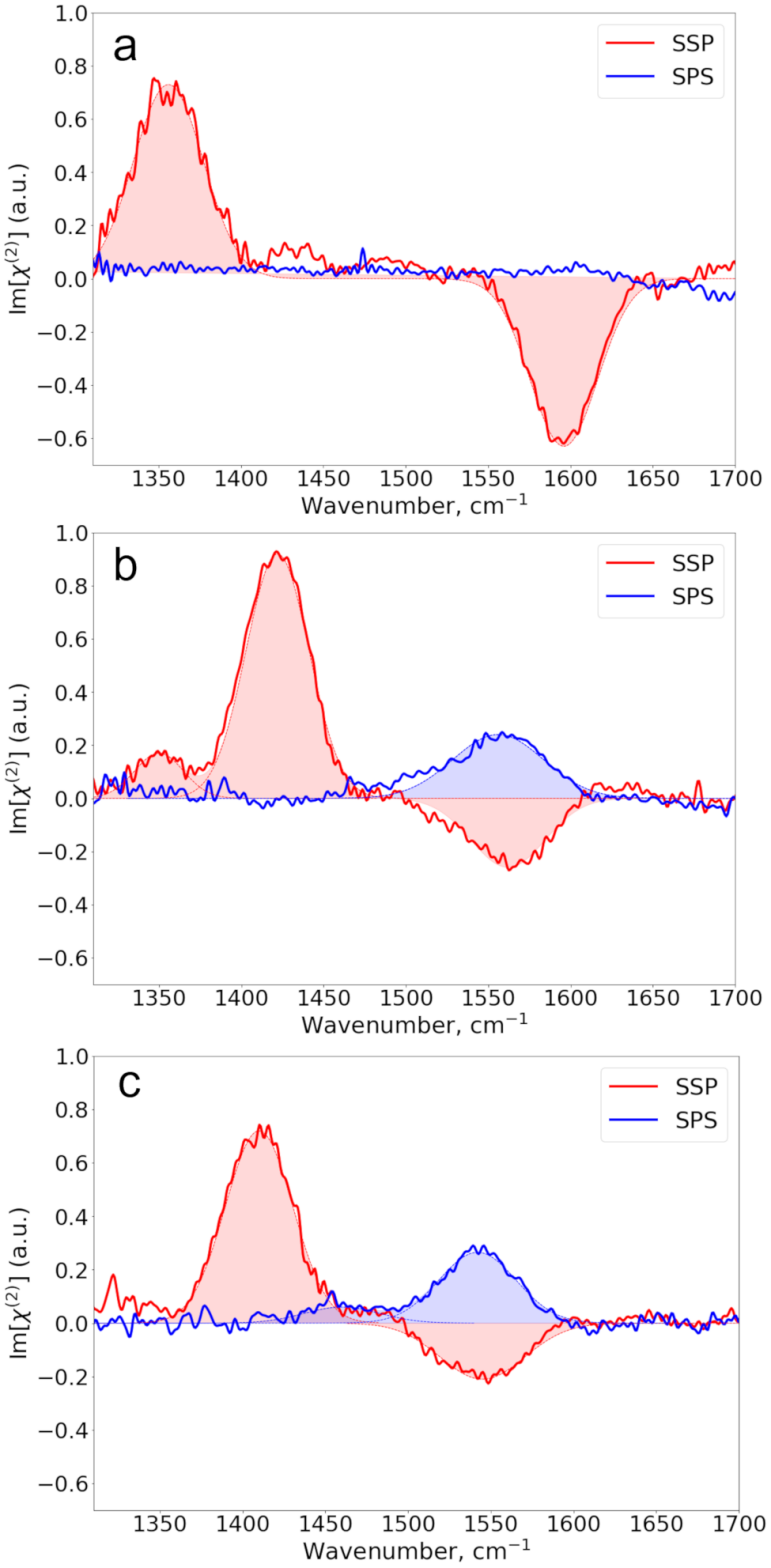
SSP and SPS Im[χ^(2)^] spectra of (a) 4.5 m sodium formate, (b) 2.5 m sodium acetate, and (c) 1 m sodium propionate. The solid lines represent the experimental spectra, while the filled dashed lines represent Gaussian fits to the data.

In Figure 3 we show Im[χ^(2)^] spectra of formate, acetate, and propionate D_2_O solutions, measured in SSP and SPS polarization configurations. In Figure 3a it is seen that the SSP spectrum of formate shows a positive band at ∼1355 cm^–1^ and a negative band at ∼1590 cm^–1^, corresponding to the ν_s_ and ν_as_ vibrations of the carboxylate group, respectively. The SPS spectrum of formate does not show a significant signal in this frequency region, in agreement with the results of our previous study.^39^ For sodium acetate (Figure 3b), we also observe a positive band at ∼1415 cm^–1^, corresponding to ν_s_, and a negative band centered at ∼1565 cm^–1^, corresponding to ν_as_ in the SSP spectrum. The band centered at ∼1350 cm^–1^ corresponds to the  vibration. In contrast to formate, the SPS spectrum of acetate does show a significant signal, in the form of a clear positive peak near 1560 cm^–1^, corresponding to the ν_as_ vibration. Finally, in Figure 3c the SSP-spectrum of sodium propionate shows a positive band at ∼1415 cm^–1^ and a negative band at ∼1550 cm^–1^ that are assigned to the ν_s_ and ν_as_ vibrations, respectively. The SPS spectrum shows a strong positive peak at 1550 cm^–1^ corresponding to the ν_as_ vibration and an additional weak positive peak at ∼1465 cm^–1^ that corresponds to the  vibration. For acetate and propionate, the absolute amplitudes of the bands corresponding to the ν_as_ vibration are very similar in the SSP and SPS spectra, whereas for formate, the ν_as_ band is much stronger in SSP than in SPS. As follows from eq 5, these findings indicate that the tilt angle of the carboxylate group of formate is very different from that of acetate and propionate and that the tilt angles of acetate and propionate are quite similar.

**Figure 4:**
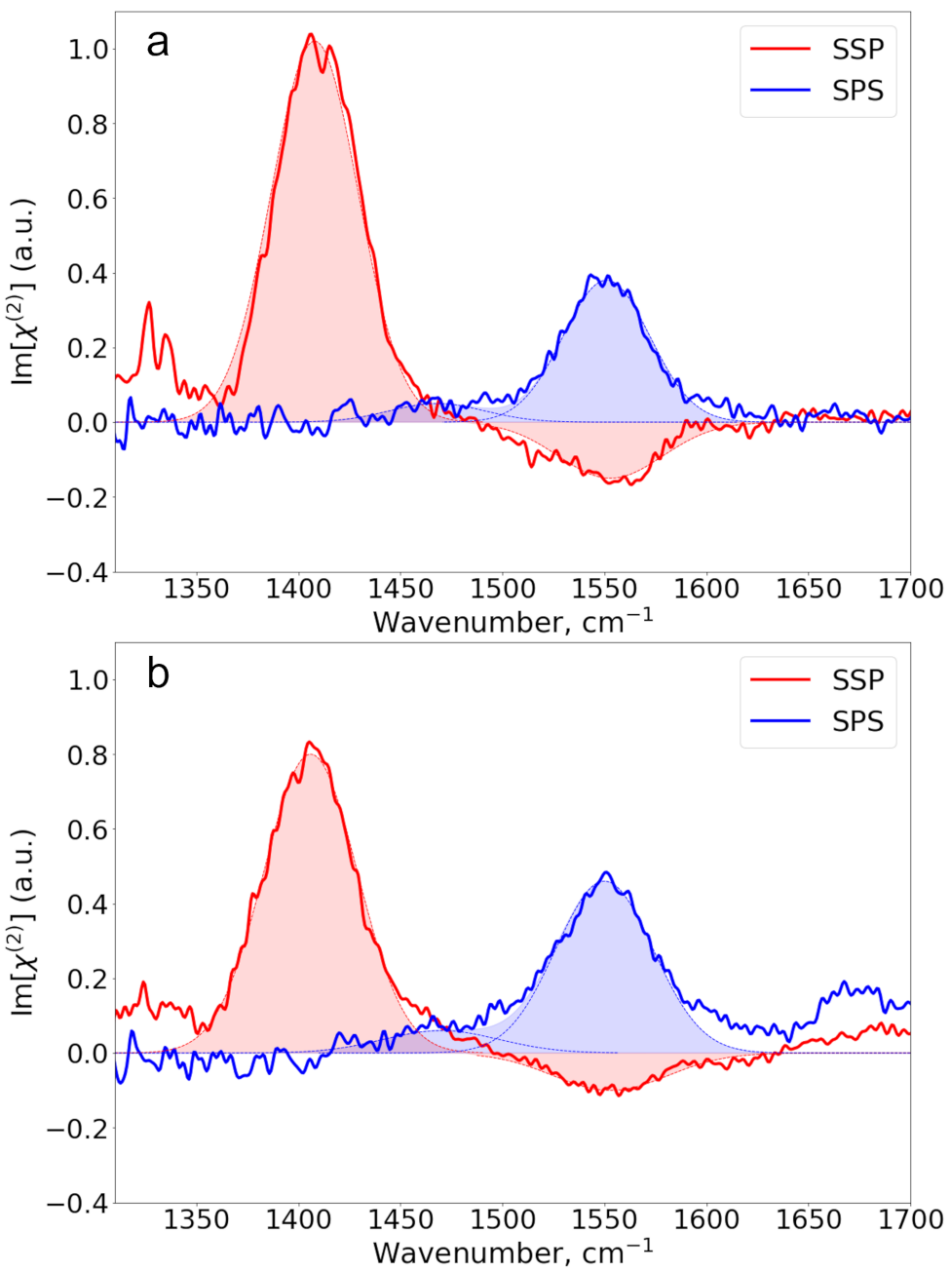
SSP and SPS Im[χ^(2)^] spectra of (a) 0.2 m sodium hexanoate and (b) 0.04 m sodium octanoate. The solid lines represent experimental spectra, while the filled dashed lines represent the Gaussian fit to the data.

To further investigate the dependence of Im[χ^(2)^] spectra on the aliphatic chain length, we also measured the Im[χ^(2)^] spectra of hexanoate and octanoate solutions, as shown in Figure 4. The spectra of the two ions are quite similar, and the assignment of the observed bands is the same as for propionate. The absolute amplitude of the ν_as_ band is lower in the SSP spectra than in the SPS spectrum, which indicates that the carboxylate group has a different orientation compared to that of acetate and propionate.

**Figure 5:**
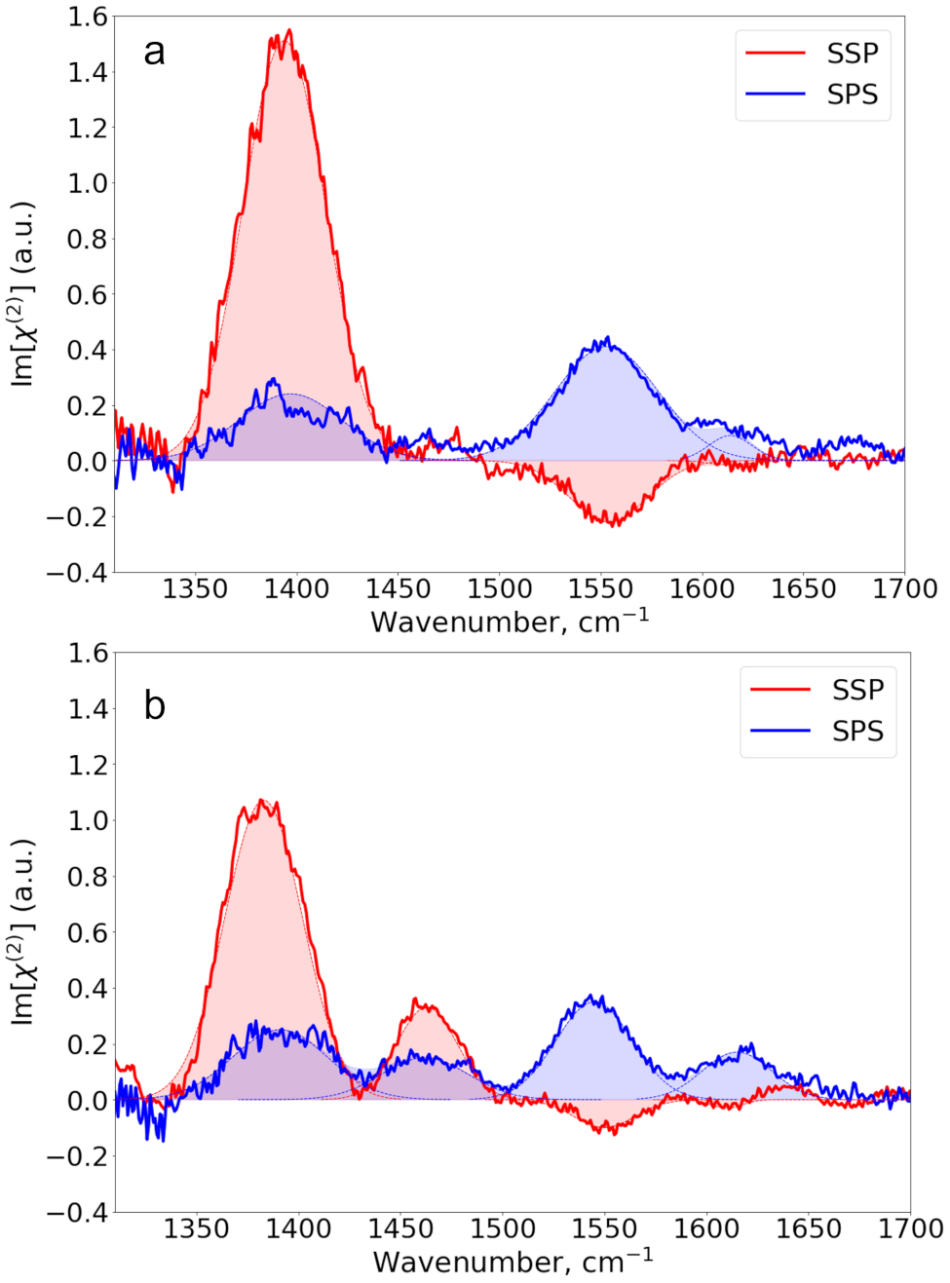
SSP and SPS Im[χ^(2)^] spectra of (a) 0.3 m sodium benzoate and (b) 0.075 m sodium 2-naphthoate. The solid lines represent experimental spectra, while the filled dashed lines represent the Gaussian fit to the data.

Finally, we examine the Im[χ^(2)^] spectra of the aromatic ions benzoate and 2-naphtoate as shown in Figure 5. The SSP spectrum of sodium benzoate shows a positive band at ∼1390 cm^–1^ and a negative band centered at ∼1555 cm^–1^, corresponding to the ν_s_ and ν_as_ vibrations, respectively. The SPS spectrum of benzoate shows two bands at similar frequencies, both with positive amplitude. In addition, we observe a weak positive band at ∼1610 cm^–1^. Following the assignment of the FTIR absorption spectra, we assign this band to a vibration of mixed character involving the ν_as_ vibration of the carboxylate group and ring vibrations of the benzoate ion. The Im[χ^(2)^] spectra of the 2-naphthoate ion contain multiple bands. Similarly to the benzoate ion, the band centered at ∼1390 cm^–1^ corresponding to the ν_s_ vibration is positive in both the SSP and SPS spectrum, while the band centered at ∼1555 cm^–1^ corresponding to the ν_as_ vibration is negative in the SSP spectrum and positive in the SPS spectrum. The other bands are assigned to ring modes of the 2-napthoate ion. For both benzoate and 2-naphthoate, the ν_as_ band has a higher absolute amplitude in the SPS spectrum than in the SSP spectrum.

To obtain a quantitative determination of the tilt angle of the different carboxylate ions, we fit the bands of the Im[χ^(2)^] spectra with Gaussian functions. As the widths of the bands measured in SSP and SPS polarization combinations are similar, the ratio of the band areas is well-represented by the ratio of their amplitudes. Using the extracted amplitudes we determine the |Im /Im[]| ratio. This ratio is shown in Table 1.

**Table 1:** Parameters Extracted from the Analysis of Im[χ^(2)^] Spectra

Carboxylate	ν_as_, cm^–1^	ν_s_, cm^–1^	|Im /Im[]|	|Im[]/Im[]|	(β_aac_ + β_bbc_)/β_ccc_	β_aca_/β_ccc_
Formate	1595	1350	>13	0.92 ± 0.06	2.0 ± 0.2	2.0 ± 0.2
Acetate	1565	1415	1.16 ± 0.19	0.3 ± 0.03	2.0 ± 0.2	1.2 ± 0.2
Propionate	1550	1415	0.85 ± 0.15	0.31 ± 0.04	2.0 ± 0.2	1.3 ± 0.2
Hexanoate	1550	1410	0.42 ± 0.09	0.16 ± 0.03	2.0 ± 0.2	1.2 ± 0.2
Octanoate	1550	1410	0.23 ± 0.06	0.13 ± 0.04	2.0 ± 0.2	1.2 ± 0.2
Benzoate	1555	1390	0.57 ± 0.08	0.14 ± 0.02	0.5 ± 0.2	0.7 ± 0.2
2-Naphthoate	1555	1390	0.31 ± 0.09	0.1 ± 0.02		

**Figure 6:**
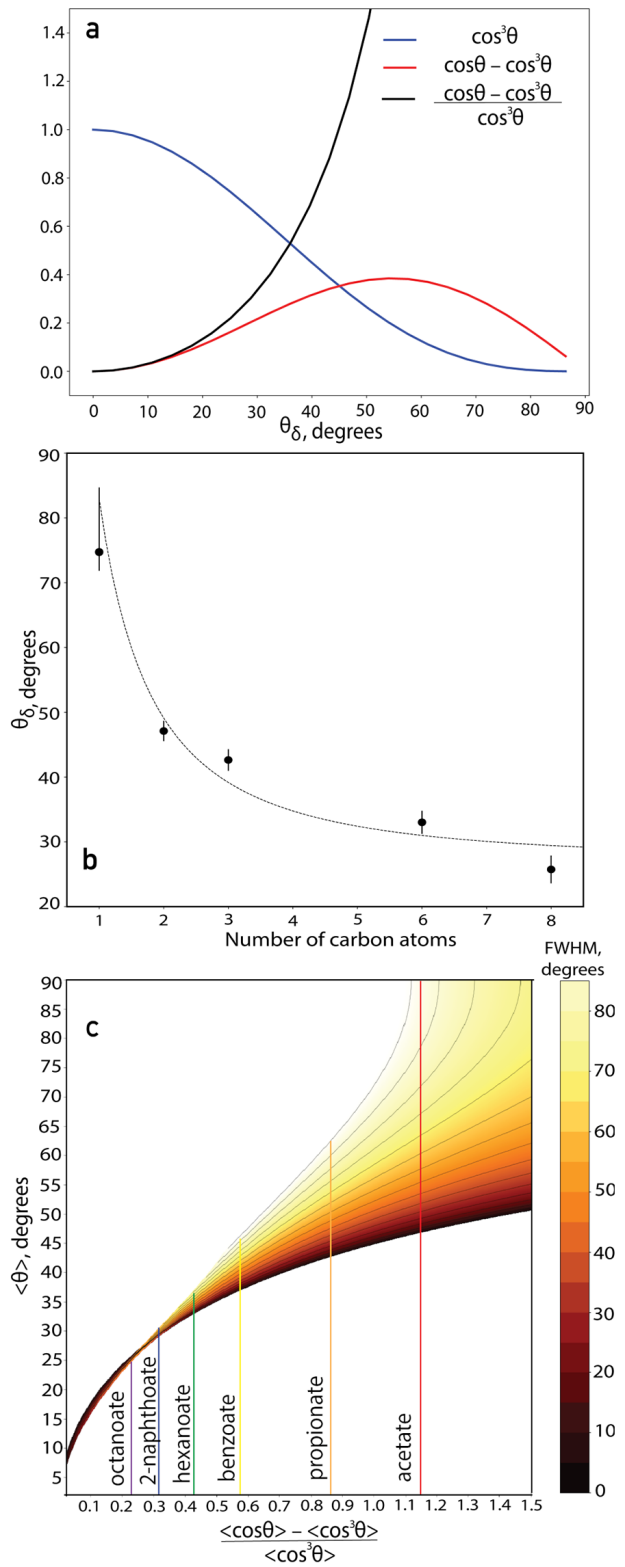
(a) Calculated dependence of the angular terms for SSP and SPS polarization combinations and the Im /Im  ratio assuming a δ-distribution of the tilt angle θ. (b) Tilt angle θ_δ_ extracted for aliphatic carboxylates with different chain lengths assuming a δ-distribution of the tilt angle θ. (c) Dependence of the average angle < θ > on the Im /Im  ratio obtained by integration over the Gaussian angular distribution described in the text. The FWHM of the distribution is reflected in the colors of the contour plot, and the colored solid lines correspond to the carboxylates under study: acetate (red), propionate (orange), benzoate (yellow), hexanoate (green), 2-naphthoate (blue), and octanoate (purple).

We first assume a δ angular distribution and calculate the angles ⟨θ⟩ = θ_δ_ using this assumption and eq 5. The dependence of the angular terms on the tilt angle on the right-hand side of eq 5 and their ratio are shown in Figure 6a.

For formate, it is not possible to determine the amplitude of Im[χ^(2)^] in the SPS spectrum, as this amplitude is close to 0. Considering the dependence of the angular term in the SPS-polarization combination, we conclude that this ion has a large tilt angle, close to 90°. However, the fact that we do observe a clear response in the SSP polarization combination shows that the angle must be smaller than 90°; otherwise, this response should also have vanished. If we estimate the minimal detectable relative Im[χ^(2)^] value to be 0.05, we obtain an absolute |Im /Im[]| ratio > 13, which implies a tilt angle θ_δ_ > 75°.

For acetate and propionate, the values of θ_δ_ are 47° ± 2° and 42° ± 3°, respectively, meaning that these angles are similar. The long-chain carboxylates are less tilted: θ_δ_ = 33° ± 3° for hexanoate and θ_δ_ = 26° ± 3° for octanoate. For the aromatic carboxylates, we extract θ_δ_ = 37° ± 3° and θ_δ_ = 29° ± 4° for benzoate and 2-naphthoate, respectively. The tilt angles of the aliphatic carboxylates are presented in Figure 6b as a function of the number of carbon atoms. It is clearly seen that θ_δ_ decreases with an increase in alkyl chain length.

Using a δ angular distribution for the tilt angle is not a realistic approximation. Therefore, we also analyzed the orientation of the ions, including a Gaussian angular distribution function with a central angle and a certain width. To investigate how the ratio of eq 5 depends on the central angle and the width of the Gaussian distribution, we integrated the angular terms in the ratio of eq 5 as well as the angle θ over Gaussian distribution functions with different central angles θ_c_ and values for the FWHM of the Gaussian varying between 0° and 90°. It should be noted that these distribution functions are not symmetric, as the angle θ can only have positive values. Moreover, the θ values in the distribution near zero (i.e., perpendicular to the surface) will negligibly contribute because of the sin(θ) term in the integration over spherical coordinates. The details of this calculation can be found in the Supporting Information.

Combining the calculated dependencies of  ratio and ⟨θ⟩ on the parameters of the distribution, we obtain the dependence of the average tilt angle ⟨θ⟩ on the  ratio and FWHM of the distribution, which we show in Figure 6c. We find that, for ratios less than 0.4, the ⟨θ⟩ value is not very sensitive to the distribution width, and thus, for octanoate, 2-naphthoate, and hexanoate, we conclude that the average tilt angle is very close to θ_δ_, irrespective of the width of the angular distribution. For ratios greater than 0.4, which is the case for benzoate, propionate, and acetate, the average tilt angle ⟨θ⟩ has θ_δ_ as its minimum value and becomes larger when the width of the angular distribution increases. This effect becomes more pronounced with an increase in ratio.

We further investigated the relations between the hyperpolarizability components corresponding to the vibrations associated with the carboxylate group. As follows from equation 4, based on the absence of the band corresponding to the ν_s_ vibration in the SPS spectra of the aliphatic species, we conclude that β_aac_ + β_bbc_ ≈ 2β_ccc_. For benzoate and 2-naphtoate the ν_s_ vibration is observed in the SPS spectra. Using the observed ratio and the values of ⟨cos θ⟩ and , as determined from the responses measured for the ν_as_ vibration in SSP and SPS configurations, we can determine the ratio (β_aac_ + β_bbc_)/β_ccc_ for benzoate, and we obtain for this ratio a value of 0.5 ± 0.2. We do not perform a similar analysis for the 2-naphthoate ion because the character of the ν_s_ vibration is likely smeared out over many different bands, as shown in Figure 2. We also determined the ratio between β_aca_ and β_ccc_ using the |Im /Im[]| ratio that can be obtained by dividing equation 1 by equation 3. The results of the analysis are summarized in Table 1, and details of the calculations are given in the Supporting Information.

## Discussion

In previous studies of long-chain fatty acids adsorbed at the water–air interface,^14,45^ only the ν_s_ vibrational band of the carboxylate group was observed, and the ν_as_ band was not detected. A similar observation was made for carboxylate ions adsorbed on fluorite^43^ and on nanoceria surfaces.^46^ These observations contrast with the present work; the SSP Im[χ^(2)^] spectrum shows clear responses of both the ν_s_ and ν_as_ vibrations of the carboxylate group. The response of the ν_as_ band in the SSP Im[χ^(2)^] spectrum is determined by the magnitude of the hyperpolarizability component β_aca_ and the orientation of the ion. From the angular terms of equation 1 it follows that the ν_as_ vibration can only be observed if, for the carboxylate ions investigated in this work, the net transition dipole of the vibration is not parallel to the surface (⟨θ⟩ ≠ 0°). In the cited previous studies long carboxylate ions in packed monolayers and carboxylate ions adsorbed to solid surfaces in a bidentate manner were studied. In these cases, the tilt angle of the main axis will be close to zero, which means that the transition dipole moment of the ν_as_ vibration is likely oriented close to parallel to the surface, which likely explains why this vibration was not observed in the SFG spectrum. Interestingly, for formate adsorbed to a fluorite surface, the ion has been shown to have a nonzero tilt angle, but nevertheless, the ν_as_ band was still not observed.^43^ This latter result was attributed to the low probability of Raman transition corresponding to the ν_as_ vibration, which enters as a factor in the expression for the β_aca_ component. Indeed, the Raman response of the ν_as_ vibration of formate in aqueous solutions and in solid salts has been observed to be quite small.^41^ An advantage of the technique of HD-VSFG used in the present study is that, with this technique, the complex χ^(2)^ is measured directly, whereas in previous intensity SFG experiments, the measured response was proportional to |χ^(2)^|^2^. Weak resonances like that of the ν_as_ vibration are much more easily distinguished in the complex χ^(2)^ spectrum than in the |χ^(2)^|^2^ spectrum, as this latter spectrum is dominated by the stronger resonances, i.e., ν_s_, and also usually gets further complicated by interference effects between different resonances and between resonances and a nonresonant background.

In the SPS spectra of propionate, hexanoate, and octanoate a weak band centered at ∼1465 cm^–1^ is observed that is assigned to the  band. Interestingly, this band has been observed before in the SSP spectrum of propionate, while for octanoate, no clear response was detected when the ions were adsorbed to a fluorite surface.^43^ This difference likely also originates from a difference in the orientation of the carboxylate ion at the surface. The observation of  also requires that the net orientation of the transition dipole of the  vibration significantly differs from parallel to the surface.

Our results show that small carboxylate ions have their carboxylate groups significantly tilted at the water–air interface. Formate ion constitutes a special case, showing a very large tilt angle. As formate does not have a clear hydrophobic part like the other carboxylates, it is likely that its orientation at the water surface is not driven by preferential dehydration of its hydrophobic part. In accordance with this notion, previous MD studies point to a very low surface activity of formate and a preference for bulk-like solvation of this ion.^47^ Recently, Yu et al. applied HD-VSFG combined with ab inito molecular dynamics (AIMD) simulations to investigate the orientation of formic acid (the protonated form of formate).^38^ In this work, the tilt angle for the C–H bond of the ion that coincides with the tilt angle defined in the present study was found to be ∼56°. This implies that the protonated carboxylic acid group is less tilted compared to the deprotonated carboxylic group of the formate. This can be explained from the fact that the charge distribution has a more localized character and is more asymmetric in the neutral formic acid molecule than in the formate ion.

For acetate and propionate, the tilt angles are smaller than those for formate, which can be well-explained by the presence of a methyl and ethyl group in their molecular structure that causes the ions to be more hydrophobic than formate. For both ions, θ_δ_ ≈ 45°. Interestingly, in MD simulations of acetate at the water–air interface, it was found that the orientational distribution would show two maxima.^47^ The first maximum is around 0° and corresponds to ions pointing with their carboxylate group maximally into the bulk, while the second maximum, with a much smaller amplitude, would correspond to ions pointing into the bulk with their methyl groups. A comparison with our work is difficult because of the completely different form of the orientational distribution functions, assumed to be Gaussian versus doubly peaked. In previous studies it was found that the spectroscopic results could be well-described with a Gaussian orientational distribution.^48,49^ However, it would be of high interest if more detailed information on the shape of the orientational distribution function of various carboxylate ions could be obtained, for instance, by combining spectroscopic results with molecular dynamics simulations. Such a combined approach involving VSFG measurements has recently been successfully used to elucidate the orientational distribution of formic acid at a water–air interface.^38^ An interesting finding is that the tilt angles of the longer-chain hexanoate and octanoate ions are quite close to those of the 2-naphthoate ion. This is most likely because the sizes of the hydrophobic parts of these ions are very similar. For benzoate ion, the tilt angle is slightly larger (θ_δ_ = 37°), which again can be well-explained from the smaller size of its hydrophobic part. The benzoate ion has a smaller tilt angle than acetate and propionate but larger than those of hexanoate and octanoate and thus takes an intermediate position between long-chain and small-chain carboxylates.

The effect of including a nonzero width of the angular distribution function on the obtained values of ⟨θ⟩ strongly depends on the experimental |Im /Im[]| ratios. For small ratios and θ_δ_ < 30°, the effect is negligible; hence, ⟨θ⟩ has to be similar to θ_δ_, irrespective of the width of the angular distribution, which applies to compounds with large substituents such as long-chain carboxylates. However, smaller ions that have a larger ⟨θ⟩ require more information on the orientation distribution function for an unambiguous determination of ⟨θ⟩, as a broad range of ⟨θ⟩ and distribution widths yield the same |Im[]/Im[]| ratio as determined from the VSFG experiments.

Finally, based on the information related to the orientational distribution, we extract ratios of hyperpolarizability components for the carboxylate ions, namely, (β_aac_ + β_bbc_)/β_ccc_ and β_aca_/β_ccc_. For the aliphatic species, (β_aac_ + β_bbc_)/β_ccc_ ≈ 2 was extracted. Interestingly, a previous theoretical study of the hyperpolarizability components for the CH_2_ group, which like the carboxylate group possesses *C*_2*v*_ symmetry, yielded β_bbc_ = 0 and β_aac_ = 2β_ccc_,^50^ which is in close agreement with the present results for the carboxylate ion. In view of the similarity of the obtained (β_aac_ + β_bbc_)/β_ccc_ ratios for small and intermediate chain length carboxylates, this result can likely be generalized and used for future studies of orientational properties of different species in which the −COO^–^ group is attached to an sp^3^-hybridized carbon atom such as deprotonated residues of proteins and other biomolecules. For benzoate we obtain quite different results, namely, (β_aac_ + β_bbc_)/β_ccc_ ≈ 0.6. This difference can probably be explained by the interaction of the π-electrons of the carboxylate group with the π-electrons of the highly polarizable aromatic ring.

## Conclusions

We studied the orientation of different carboxylate anions at the water–air interface with heterodyne-detected vibrational sum frequency generation (HD-VSFG) experiments. We studied the aliphatic carboxylate anions formate, acetate, propionate, hexanoate, and octanoate and the aromatic carboxylate anions benzoate and 2-naphthoate. We probed the ν_s_ and ν_as_ stretching vibrations of the carboxylate group in the 6 μm region. For all ions, we observe a clear surface response of the vibrations of the carboxylate groups and different substituents. From the ratio of the amplitudes of the responses of the ν_as_ vibration measured in SSP and SPS polarization combinations (Im /Im ), we determine the tilt angle θ_δ_ of the ions at the water/air interface assuming a δ angular distribution. We find that increasing the size of the hydrophobic part of the ion leads to a decrease in the tilt angle of the carboxylate group of ions. Formate ion has a large θ_δ_ (>75°), while acetate and propionate have a θ_δ_ of ∼47 ± 2° and ∼42 ± 3°, respectively. For hexanoate and octanoate, we obtain tilt angles θ_δ_ of 33° ± 3° and θ_δ_ = 26° ± 3°, respectively. For the aromatic carboxylates, we extract θ_δ_ = 37° ± 3° and θ_δ_ = 29° ± 4° for benzoate and 2-naphthoate, respectively.

We further investigated the effect of the width of the angular distribution, assuming this distribution to be Gaussian. By integrating the cosine terms over the distribution, we obtain the relation between the average tilt angle ⟨θ⟩ and the Im /Im  ratio, as is determined from the experiments. For the larger aliphatic carboxylate anions and naphthoate, we find that the average tilt angle ⟨θ⟩ is independent of the width of the Gaussian angular distribution and thus equal to θ_δ_. For acetate, proprionate, and benzoate, the average tilt angle ⟨θ⟩ that follows from the measured ratio Im /Im , has θ_δ_ as its minimum value and becomes larger with increasing width of the angular distribution. Finally, using the additional information encoded in the amplitudes of the peaks corresponding to the ν_s_ vibration, we obtain (β_aac_ + β_bbc_)/β_ccc_ and β_aca_/β_ccc_ ratios for the carboxylate group. The (β_aac_ + β_bbc_)/β_ccc_ ≈ 2 for aliphatic ions, which agrees with previous estimations for CH_2_ groups. For the benzoate ion, we find (β_aac_ + β_bbc_)/β_ccc_ ≈ 0.5. This difference can probably be explained from the interaction of the π-electrons of the carboxylate group with the π-electrons of the highly polarizable aromatic ring. For formate, we get a ratio β_aca_/β_ccc_ of ∼2, which is larger than the ratio of ∼1.2 obtained for the other aliphatic ions and the ratio of ∼0.7 obtained for the benzoate ion.

## Supporting Information Available

Dependence of the Im /Im  ratio on the Fresnel coefficients and the experimental geometry, integration of angular terms over angular distribution, determination of the relations between the hyperpolarizability components (PDF)
