# Supplementary material for: Molecular Orientation of Carboxylate Anions at the Water–Air Interface Studied with Heterodyne-Detected Vibrational Sum-Frequency Generation
Source: J Phys Chem B. 2023 Mar 14;127(20):4544–53. doi: 10.1021/acs.jpcb.2c08992 (PMC10226120; doi:10.1021/acs.jpcb.2c08992)
Supplement: Supplementary file 1 — jp2c08992_si_001.pdf [file jp2c08992_si_001.pdf]

Molecular Orientation of Carboxylate Anions at the Water/Air Interface Studied with Heterodyne-Detected Vibrational Sum-Frequency Generation. Supporting Information.

Alexander A. Korotkevich,* Carolyn J. Moll, Jan Versluis, and Huib J. Bakker

AMOLF, Ultrafast Spectroscopy, 1098XG, Science Park 104 Amsterdam, Netherlands

E-mail: A.Korotkevich@amolf.nl

Dependence of the Im$[\chi^{(2)}_{SSP,\nu_{as}}]$/Im$[\chi^{(2)}_{SPS,\nu_{as}}]$ ratio on the Fresnel coefficients and the experimental geometry

The measured $\chi^{(2)}$ value is related to the $\chi^{(2)}$ in laboratory coordinate system according to:[1]

$$\chi^{(2)}_{SSP} = L_{yy}(\omega_{SF})L_{yy}(\omega_{vis})L_{zz}(\omega_{IR})sin\varphi_{IR}\chi^{(2)}_{xxz} \quad (1)$$

$$\chi^{(2)}_{SPS} = L_{yy}(\omega_{SF})L_{zz}(\omega_{vis})L_{yy}(\omega_{IR})sin\varphi_{vis}\chi^{(2)}_{xzx} \quad (2)$$

Dividing equation 1 by equation 2 yields a dependence of the ratio of the SSP and SPS signals on $L_{yy}(\omega_{vis})L_{zz}(\omega_{IR})\sin\varphi_{IR}/(L_{zz}(\omega_{vis})L_{yy}(\omega_{IR})\sin\varphi_{vis})$

The incidence angles of ω_{vis}, $\varphi_{vis} \approx 50°$ and ω_{IR}, $\varphi_{IR} \approx 55°$ are accounted for in the calculations of the tilt angles according to equation 5 of the main text.

We investigate the dependence of the Fresnel coefficient accounting for the change of n_{vis} and n_{IR} upon dissolving sodium carboxylates in water. We calculate the Fresnel coefficients using the three-layer model combined with the modified Lorentz model to determine the interfacial refractive index n'.[1,2]

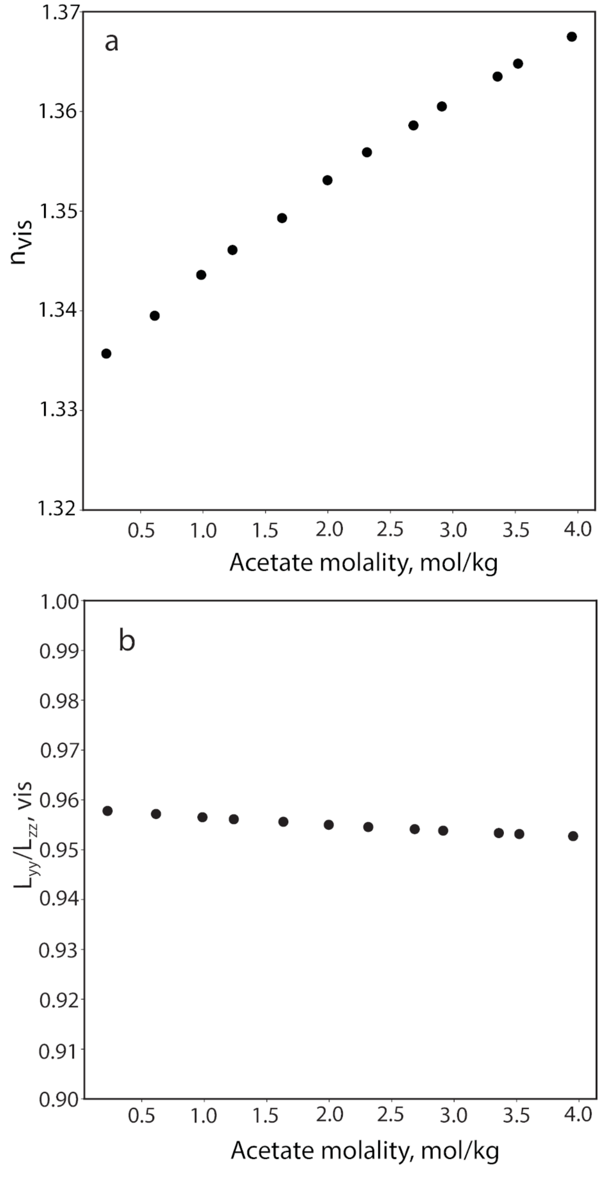

Figure S1: a. Dependence of the refractive index n_{vis} at ω_{vis} on the concentration of sodium acetate b. Dependence of $L_{yy}(\omega_{vis})/L_{zz}(\omega_{vis})$ on the concentration of sodium acetate

For n_{vis}, we used refractometry data of sodium acetate solutions obtained from previous work.[3] As can be seen from Figure S1a, n_{vis} shows a linear increase with concentration.

As can be seen from Figure S1b, this increase leads to <1% change in the ratio of Fresnel coefficients. Hence, the Im[$\chi^{(2)}$] spectra of solutions with different concentrations of acetate do not show a strong variation of the Fresnel coefficient at ω_{vis}.

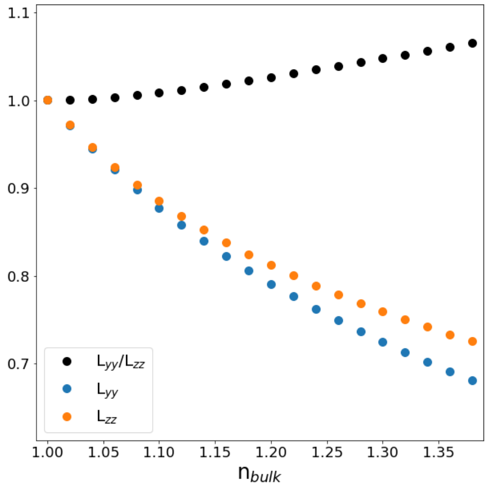

Figure S2: Dependence of L_{zz}, L_{yy} and the L_{zz}/L_{yy} ratio on the bulk refractive index

The concentration dependence of the Fresnel coefficients near ω_{IR} has been investigated before.[4] The absorption of infrared light by carboxylate ions causes dispersion of the refractive index n_{IR}, and the magnitude of this dispersion increases with increasing carboxylate concentration. This dispersive effect primarily leads to a shift of the maximum of the spectrum of the generated sum-frequency light, and hardly affects the amplitude of the band. In Figure S2 we show the dependence of the ratio $L_{zz}(\omega_{IR})/L_{yy}(\omega_{IR})$ on the bulk refractive index. As can be seen, the change of the ratio $L_{zz}(\omega_{IR})/L_{yy}(\omega_{IR})$ induced by the change of the refractive index does not exceed 5%. The value of the ratio $L_{yy}(\omega_{vis})L_{zz}(\omega_{IR})/(L_{zz}(\omega_{vis})L_{yy}(\omega_{IR}))$ value will thus be close to 1 at all infrared frequencies, as can be seen from Figures S1 and S2. Hence, we conclude that the variation of $L_{yy}(\omega_{vis})L_{zz}(\omega_{IR})/(L_{zz}(\omega_{vis})L_{yy}(\omega_{IR}))$ with salt concentration has a negligible effect on the determination of the tilt angle based on the experimental $\mathrm{Im}[\chi^{(2)}_{SSP,\nu_{as}}]/\mathrm{Im}[\chi^{(2)}_{SPS,\nu_{as}}]$ ratio.

Integration of angular terms over angular distribution

To calculate the average values of $\langle\theta\rangle$, $\langle cos\theta\rangle$ and $\langle cos^3\theta\rangle$ an integration over the angular distribution function is required. The Gaussian distribution function is defined as:

$$F(\theta, FWHM) = exp(-\frac{1}{2}\frac{(\theta-\theta_c)^2}{(\frac{FWHM}{2\sqrt{ln2}})^2}) \tag{3}$$

where θ_c is the central tilt angle and FWHM is the full width at half maximum of the distribution. The average value is then calculated as follows:

$$\langle f(\theta)\rangle = \frac{\int_0^\pi d\theta f(\theta)F(\theta, FWHM)sin\theta}{\int_0^\pi d\theta F(\theta, FWHM)sin\theta} \tag{4}$$

where $f(\theta) = \theta$, $cos\theta$ or $cos^3\theta$; the denominator is used for normalization with respect to the Gaussian area. Of course, if a δ-distribution is assumed, $\langle\theta\rangle = \theta_c \equiv \theta_\delta$, $\langle cos\theta\rangle = cos\theta_\delta$, $\langle cos^3\theta\rangle = cos^3\theta_\delta$. The dependence of the angular terms assuming a delta angular distribution is presented in Figure 6b of the main text. By performing numerical integrations for θ_c and FWHM varying between 0 and 90°, we obtain the dependence of the ratio $\text{Im}[\chi^{(2)}_{SSP,\nu_{as}}]/\text{Im}[\chi^{(2)}_{SPS,\nu_{as}}]$ on θ_c and FWHM.

In Figure S3 we show the dependencies of angular terms determining the amplitude of the band corresponding to the ν_{as} in SSP (Figure S3a) and SPS (Figure S3b) spectra on the parameters of Gaussian distribution.

By dividing the dependence in Figure S3a by the dependence in Figure S3b we obtain the dependence of the $\frac{\langle cos\theta\rangle-\langle cos^3\theta\rangle}{\langle cos^3\theta\rangle}$ ratio that we show in Figure S4a. This ratio is directly related to the $\text{Im}[\chi^{(2)}_{SSP,\nu_{as}}]/\text{Im}[\chi^{(2)}_{SPS,\nu_{as}}]$ obtained from the measurements.

In Figure S4b we also show the dependence of $\langle\theta\rangle$ on the parameters of the Gaussian distribution. By combining the information displayed in Figure S4a and Figure S4b we obtain the dependence of $\langle\theta\rangle$ on the $\frac{\langle cos\theta\rangle-\langle cos^3\theta\rangle}{\langle cos^3\theta\rangle}$ ratio and the FWHM of the distribution, shown in Figure 6c of the main text.

To understand the orientational properties of the formate ion, it is important to discuss

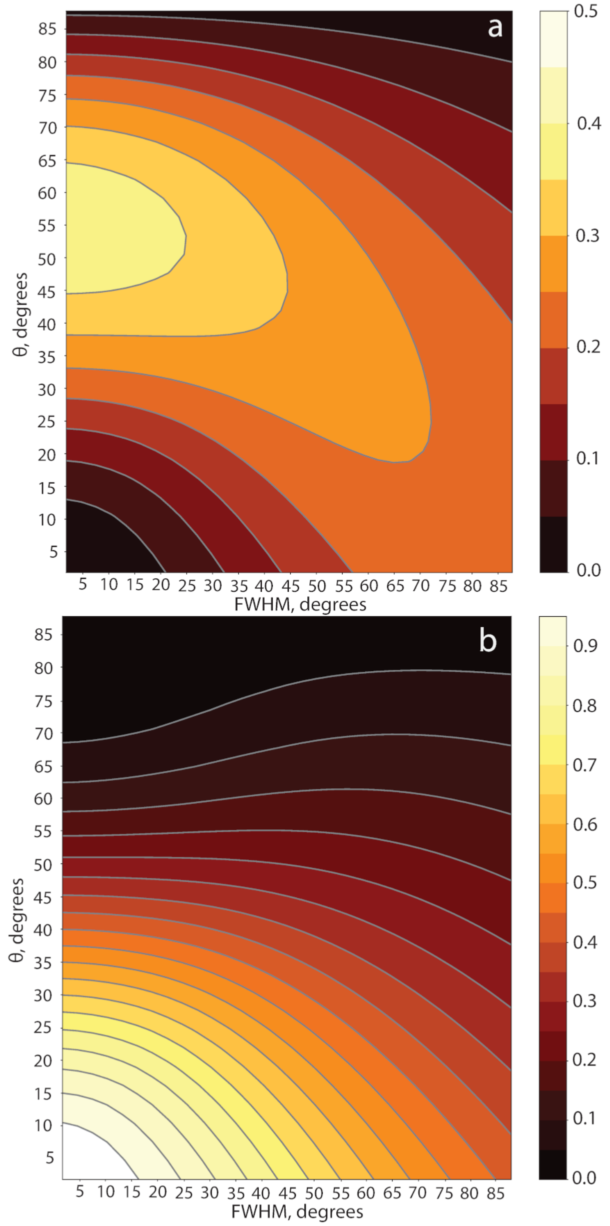

Figure S3: Dependence of a. $\langle cos\theta\rangle$ - $\langle cos^3\theta\rangle$ b. $\langle cos^3\theta\rangle$ on θ_c and FWHM of Gaussian distribution obtained by integration over Gaussian functions

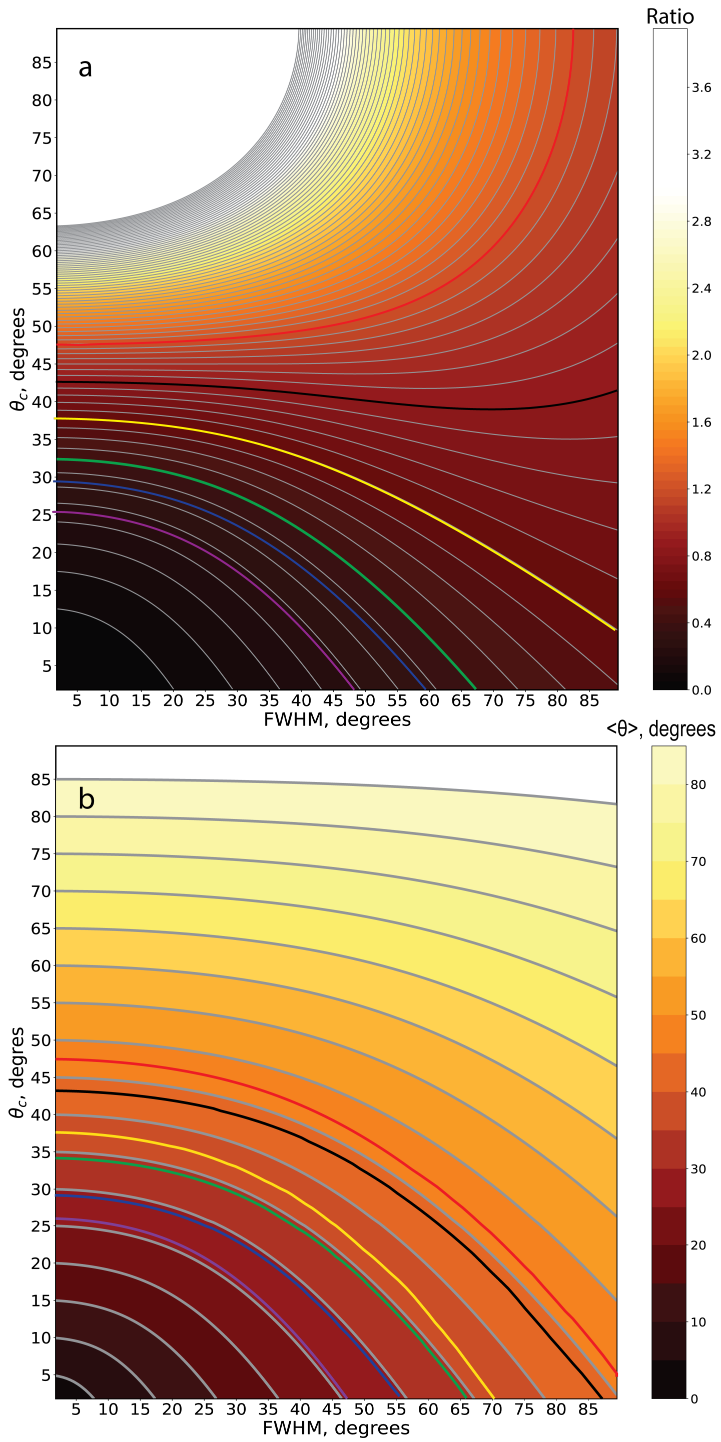

Figure S4: Dependence of a. $\frac{\langle cos\theta\rangle - \langle cos^3\theta\rangle}{\langle cos^3\theta\rangle}$ ratio and b. $\langle\theta\rangle$ on θ_c and the FWHM of the Gaussian distribution obtained from equation (4). The colored solid lines correspond to the carboxylates under study: acetate (red), propionate (black), benzoate (yellow), hexanoate (green), 2-naphthoate (blue), octanoate (purple)

the dependencies of the angular terms in Figure S3. As the amplitude of the band corresponding to the ν_{as} vibration in the SPS spectrum is very close to 0, the θ_δ must be quite large, as follows from Figure S3b. At the same time, as a strong negative response is observed in the SSP spectrum θ_δ must be smaller than 90°, as follows from Figure S3a. We estimate the smallest measurable relative Im[$\chi^{(2)}$] value to be 0.05 for SPS polarization, which yields a $\frac{\langle cos\theta\rangle - \langle cos^3\theta\rangle}{\langle cos^3\theta\rangle}$ ratio of ~ 13 and hence 75° as a lower boundary for θ_δ. Note that if a lower amplitude in the SPS spectrum was used in the calculations, only a larger tilt angle could be extracted. Furthermore, as follows from Figure S4b, with $\theta_\delta = 75°$, increasing the FWHM can only increase the $\langle\theta\rangle$, thus we conclude that $\langle\theta\rangle > 75°$ for formate.

Determination of the relations between the hyperpolarizability components

As can be noticed directly from the measurements, the band corresponding to the ν_s vibration is absent in the SPS spectra of the aliphatic carboxylates. As follows from equation 4 of the main text, this observation implies that $\beta_{aac} + \beta_{bbc} \approx 2\beta_{ccc}$ as the angular term is non-zero considering the non-zero amplitude of the band corresponding to the ν_{as} vibration in the SSP spectrum. Given that, it can be noted that the second term of equation 3 of the main text for the ν_s vibration in SSP polarization combination also must vanish and the expression thus simplifies to its first term. Hence, by dividing equation 1 of the main text by equation 3 of the main text we obtain:

$$\frac{Im[\chi^{(2)}_{SSP,\nu_{as}}]}{Im[\chi^{(2)}_{SSP,\nu_s}]} \approx -\frac{2\beta_{aca}}{\beta_{aac} + \beta_{bbc} + 2\beta_{ccc}} \frac{\langle cos\theta\rangle - \langle cos^3\theta\rangle}{\langle cos\theta\rangle} \approx -\frac{\beta_{aca}}{2\beta_{ccc}} \frac{\langle cos\theta\rangle - \langle cos^3\theta\rangle}{\langle cos\theta\rangle} \quad (5)$$

Therefore, the experimental ratio depends on both the angular distribution and the ratio of the β_{aca} and β_{ccc} hyperpolarizability components. Based on the information on the angular

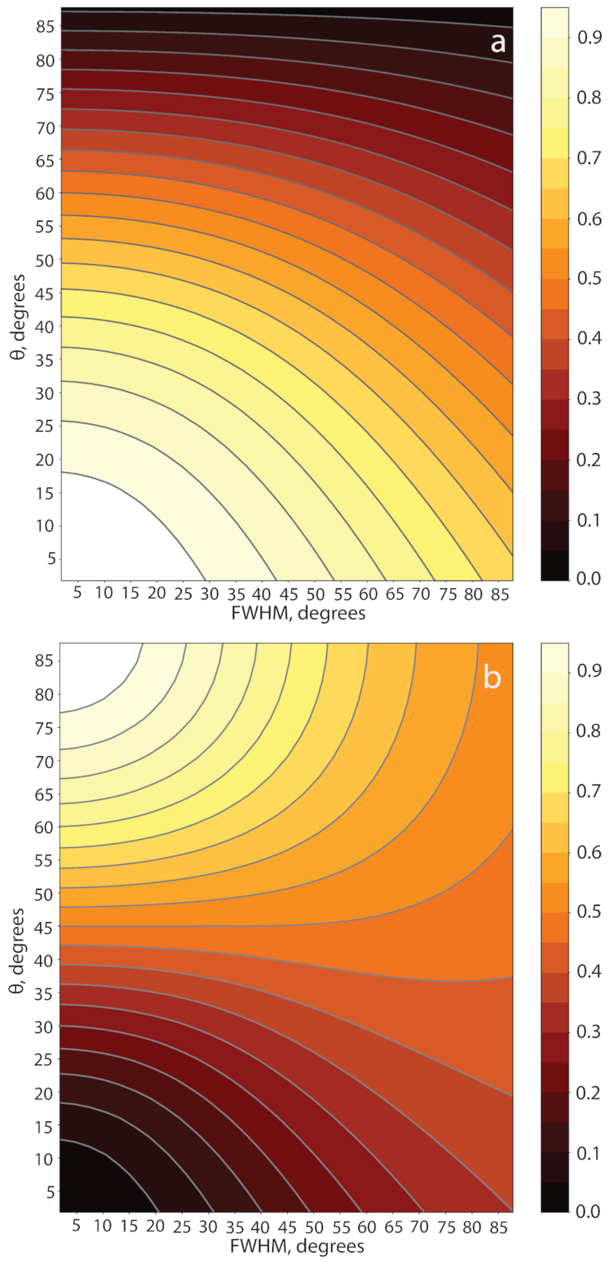

Figure S5: Dependence of a. $\langle cos\theta \rangle$ b. $(\langle cos\theta \rangle$ - $\langle cos^3\theta \rangle)/\langle cos\theta \rangle$ on θ_c and the FWHM of the Gaussian distribution obtained from equation (4).

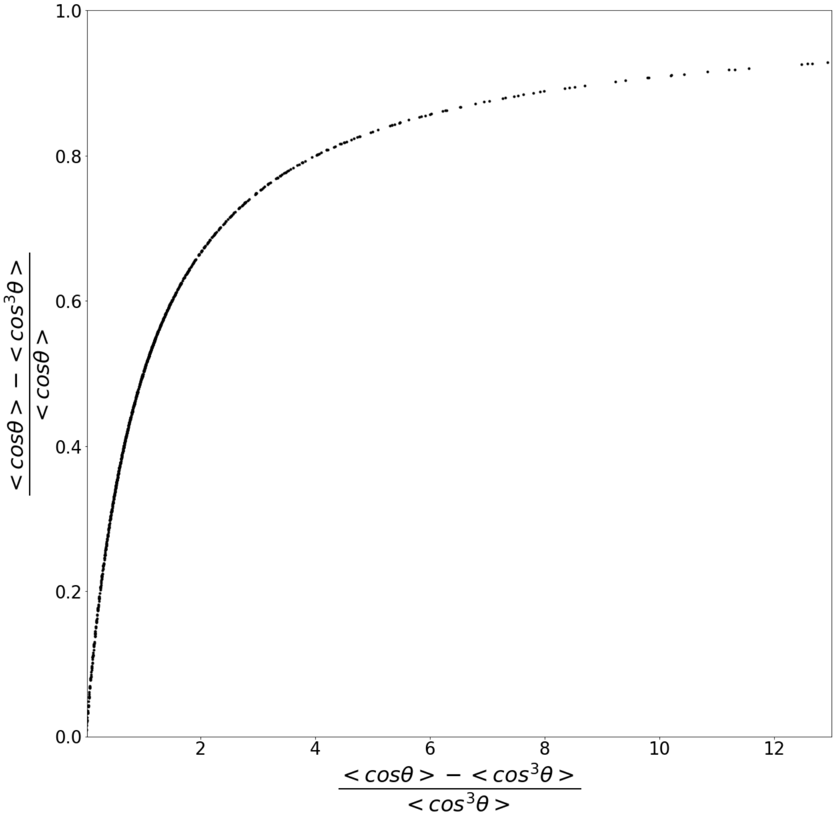

Figure S6: Dependence of $\frac{\langle cos\theta\rangle - \langle cos^3\theta\rangle}{\langle cos\theta\rangle}$ on $\frac{\langle cos\theta\rangle - \langle cos^3\theta\rangle}{\langle cos^3\theta\rangle}$ obtained from equation (4).

distribution obtained from the $\mathrm{Im}[\chi^{(2)}_{SSP,\nu_{as}}]/\mathrm{Im}[\chi^{(2)}_{SPS,\nu_{as}}]$, we can determine the value of the $\frac{\langle cos\theta\rangle - \langle cos^3\theta\rangle}{\langle cos\theta\rangle}$ entering equation 5 independently. With this information we obtain the β_{aca}/β_{ccc} for aliphatic ions.

For the aromatic species, the estimation is more elaborate as the band corresponding to the ν_s vibration is non-zero in the SPS spectrum. Dividing equation 1 of the main text by equation 4 of the main text and taking the experimental $\mathrm{Im}[\chi^{(2)}_{SSP,\nu_{as}}]/\mathrm{Im}[\chi^{(2)}_{SPS,\nu_s}]$ ratio yields:

$$\frac{Im[\chi^{(2)}_{SSP,\nu_{as}}]}{Im[\chi^{(2)}_{SPS,\nu_s}]} = -\frac{2\beta_{aca}}{\beta_{aac} + \beta_{bbc} - 2\beta_{ccc}} \approx -0.92 \tag{6}$$

Further, dividing equation 1 by equation 3 combined with the experimental $\mathrm{Im}[\chi^{(2)}_{SSP,\nu_{as}}]/\mathrm{Im}[\chi^{(2)}_{SSP,\nu_s}]$ ratio, we obtain:

$$\frac{Im[\chi^{(2)}_{SSP,\nu_{as}}]}{Im[\chi^{(2)}_{SSP,\nu_s}]} = -\frac{2\beta_{aca}}{\beta_{aac} + \beta_{bbc} + 2\beta_{ccc}} \frac{\langle cos\theta\rangle - \langle cos^3\theta\rangle}{\langle cos\theta\rangle} - \frac{2\beta_{aca}}{\beta_{aac} + \beta_{bbc} - 2\beta_{ccc}} \frac{\langle cos\theta\rangle - \langle cos^3\theta\rangle}{\langle cos^3\theta\rangle} \approx -0.14 \tag{7}$$

In this equation, the ratio of the hyperpolarizability components in the second term is known from equation 6, and the ratios of the angular terms are obtained similarly to the case of the aliphatic carboxylates, as described before. By combining equations 6 and 7, we obtain $\frac{\beta_{aac}+\beta_{bbc}}{\beta_{ccc}} \approx 0.5$ and $\frac{\beta_{aca}}{\beta_{ccc}} \approx 0.7$

References

(1) Wang, H. F.; Gan, W.; Lu, R.; Rao, Y.; Wu, B. H. Quantitative Spectral and Orientational Analysis in Surface Sum Frequency Generation Vibrational Spectroscopy (SFG-VS). *Int. Rev. Phys. Chem.* **2005**, *24*, 191–256.

(2) Zhuang, X.; Miranda, P. B.; Kim, D.; Shen, Y. R. Mapping Molecular Orientation and

Conformation at Interfaces by Surface Nonlinear Optics. *Phys. Rev. B* **1999**, *59*, 12632–12640.

(3) Bagheri, M.; Kiani, F.; Koohyar, F.; Khang, N. T.; Zabihi, F. Measurement of refractive index and viscosity for aqueous solution of sodium acetate, sodium carbonate, trisodium citrate, (glycerol + sodium acetate), (glycerol + sodium carbonate), and (glycerol + trisodium citrate) at T = 293.15 to 303.15 K and atmospheric pressure. *Journal of Molecular Liquids* **2020**, *309*, 113109.

(4) Moll, C. J.; Korotkevich, A. A.; Versluis, J.; Bakker, H. J. Molecular Orientation of Small Carboxylates at the Water/Air Interface. *Phys. Chem. Chem. Phys.* **2022**, *24*, 10134–10139.
